# Exposure to ambient air pollution during pregnancy and risk of early-onset breast cancer

**DOI:** 10.1186/s13058-025-02165-9

**Published:** 2025-11-13

**Authors:** Jessica Edlund, Wendy Yi-Ying Wu, Malin Gustafsson, Jenny Lindén, Anna Oudin, Sophia Harlid

**Affiliations:** 1 Department of Diagnostics and Intervention, Oncology, Umeå University, 901 87 Umeå, Sweden; 2 IVL Swedish Environmental Research Institute, Gothenburg, Sweden; 3 Department of Public Health and Clinical Medicine, Division for Sustainable Health, Umeå University, Umeå, Sweden; 4 Division of Occupational and Environmental Medicine, Department of Laboratory Medicine, Lund University, Lund, Sweden

**Keywords:** Breast cancer, Risk factors, Air pollution, Pregnancy

## Abstract

**Background:**

Air pollution has been linked to breast cancer risk, but previous studies have seldom considered specific exposure windows, like pregnancy. During pregnancy the breast undergoes substantial changes and exposures may have a stronger impact than if they occurred during other time periods. This study aims to identify associations between ambient air pollution exposure during pregnancy and risk of early-onset breast cancer.

**Methods:**

Using nationwide data from Swedish registers, we constructed a cohort consisting of all cancer-free women in Sweden giving birth to their first child between 1991 and 2015. Residential exposure to nitrogen dioxide (NO_2_), particulate matter < 10 μm (PM_10_) and < 2.5 μm (PM_2.5_) were modelled based on air pollution concentrations from 2019. Particulate matter between 2.5 and 10 μm (PM_coarse_) was calculated separately. Detailed data on residential addresses (including exact moving dates) were available for the entire study period, allowing for spatial variation in the exposure dataset. Mean air pollution levels were assessed at first pregnancy, last pregnancy, 35 years of age, and 2 years after the last delivery. Associations were evaluated using Cox proportional hazards regression to estimate adjusted hazard ratios (HRs) and 95% confidence intervals (CIs).

**Results:**

Among 1,019,076 women, 12,085 (1.2%) were diagnosed with breast cancer and 65.2% moved at least once between their first pregnancy and two years after their last delivery. All exposures during pregnancy periods were positively associated with breast cancer, with the highest HR observed for exposure to PM_coarse_ during the last pregnancy (HR_PMcoarse_ = 1.12 (95% CI = 1.04, 1.20) per 5 μg/m^3^ increase). The lowest HR were for NO_2_ levels estimated at 35 years of age, regardless of pregnancy status (HR_NO2_ = 1.03 (95% CI = 0.99, 1.06) per 10 μg/m^3^ increase). In analyses differentiating between invasive breast cancer and ductal carcinoma in situ, only invasive breast cancer was associated with air pollution exposure.

**Conclusions:**

In this cohort study, air pollution exposure was consistently associated with increased risk of early-onset breast cancer.

**Supplementary Information:**

The online version contains supplementary material available at 10.1186/s13058-025-02165-9.

## Background

Breast cancer is the most common malignant disease among women. Most cases occur after menopause, but the incidence of premenopausal breast cancer is increasing globally [1], particularly in higher income countries [2]. Reasons for this may include altered parity patterns with higher age at first pregnancy and fewer children [3], but this alone cannot fully explain the increased incidence [1]. Recent childbirth in itself has also been shown to associate with breast cancer risk [4], especially premenopausal [5]. This highlights the importance of pregnancy as a potential window of susceptibility as it is a period of rapid changes to both the breast tissue and the surrounding microenvironment [6].

Air pollution is one of the most well studied environmental exposures when it comes to breast cancer. It contains a mixture of particles, gases and chemicals, many of which are known to be carcinogenic and influence hormone receptors [7]. Exposure to nitrogen dioxide (NO_2_), a marker for traffic-related air pollution, has been associated with both pre- and post-menopausal breast cancer risk, with suggested higher effects for premenopausal breast cancer [8]. Another common measurement in air pollution studies is particulate matter (PM) which refers to a mixture of small airborne particles, including e.g. organic chemicals, metals, sulfate, ammonium, and nitrate [9]. So far, evidence for an association between PM exposure and breast cancer risk is uncertain and associations may vary by tumor receptor subtype and menopausal status at diagnosis [7]. Despite the suggested importance of vulnerable time periods such as pregnancy, few studies have considered these when evaluating associations between air pollution and breast cancer [6].

In the current study we focused on air pollution exposure during pregnancy and risk of early-onset breast cancer. Early-onset, or premenopausal, breast cancer is likely to be of particular interest when assessing exposures occurring during pregnancy. This is due to the fact that exposures during or near pregnancy are temporally closer to diagnosis of premenopausal breast cancer compared to postmenopausal. The study was conducted using nationwide data from Swedish registers.

## Methods

### Study population and cancer ascertainment

Using data from Swedish national registers, we conducted a cohort study including all women in Sweden with no previous cancer diagnosis, who gave birth to their first child between January 1st 1991 and December 31st 2015. Women were identified through the Swedish Medical Birth Register [10], which contains information on all deliveries in Sweden occurring since 1973. The original cohort consisted of 1 074 925 women, and after additional exclusions (Figure S1), 1 023 845 participants were retained for analyses. From these participants, we created separate data sets in order to evaluate air pollution exposures at different time points (Figure S1).

The study population was linked to multiple registers using Sweden’s unique personal identity numbers [11]. Socioeconomic variables were extracted from the Longitudinal Integrated Database for Health Insurance and Labour Market Studies (LISA) [12], family history of breast and ovarian cancer was obtained by matching to the Multi-Generation Register [13] and the Swedish Cancer Register [14], cause of death was collected from the Swedish Cause of Death Register [15], and variables used to calculate the Charlson comorbidity index were collected from the Swedish Patient Register. To assess comorbidities that might confound associations we calculated a weighted Charlson comorbidity index (wCCI) from 10 years before start of follow up to start of follow up using an algorithm adapted for Swedish register-based research [16].

Cases of breast cancer (International Classification of Diseases, 7th edition, code 170), were identified through linkage to the Swedish Cancer Register. As we did not have data on menopausal status at diagnosis for all breast cancer cases, we used early-onset breast cancer (defined as primary breast cancer diagnosis before 50 years of age) as a proxy for premenopausal breast cancer and included invasive cases, ductal carcinoma in situ (DCIS), and a small number of other non-invasive breast cancers. For a subset of the cohort (invasive breast cancer cases diagnosed from 2007 onwards) we had access to tumor characteristics, including estrogen receptor (ER) and progesterone receptor (PR) status and HER2 status, as well as menopausal status at diagnosis and whether the cancer was screening-detected or not. These data were retrieved from the Swedish National Quality Register for Breast Cancer (NKBC) [17].

## Exposure assessment

**Fig. 1:**
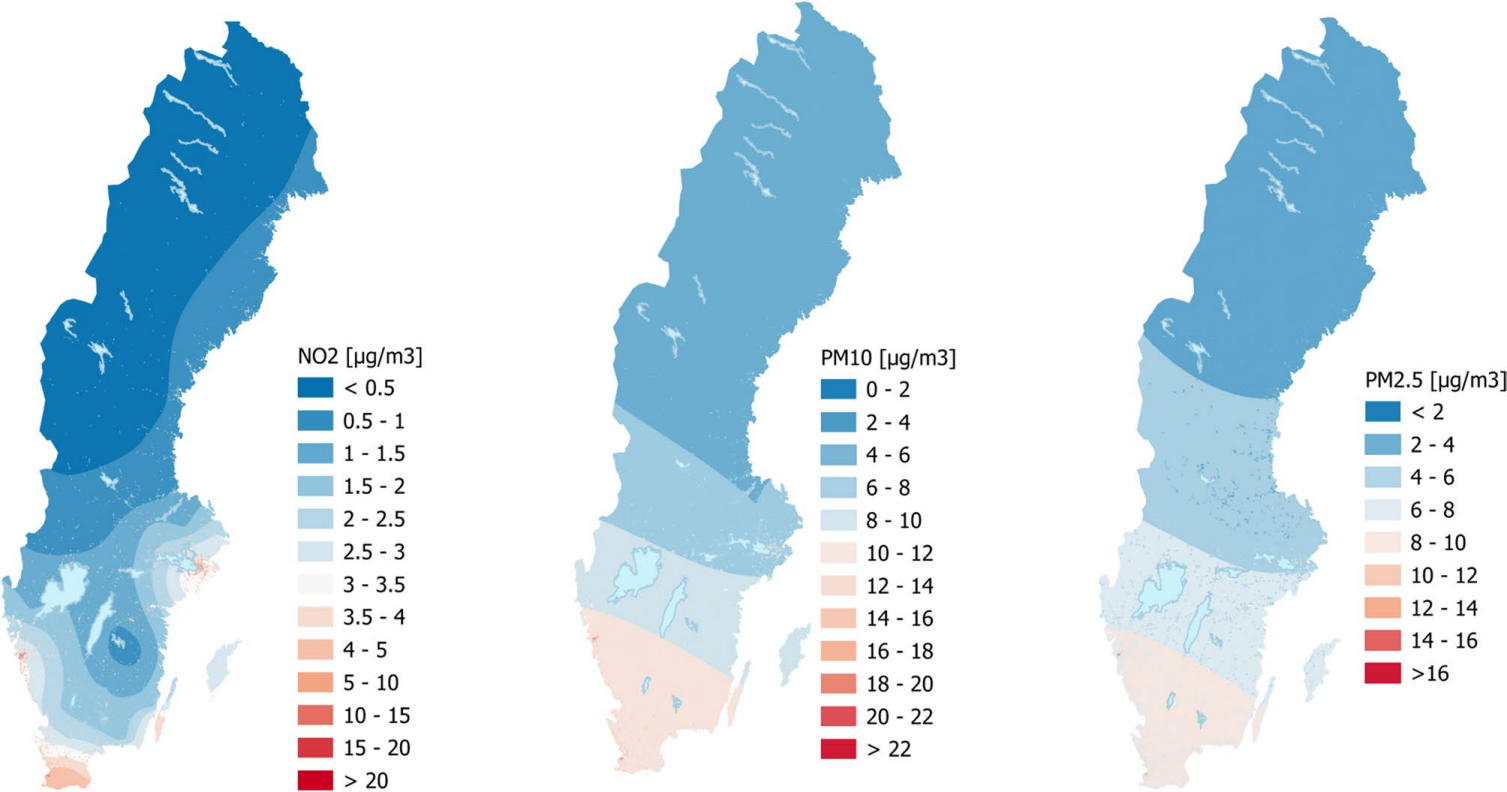
Map of Sweden with ambient air pollution (NO_2_, PM_10_, PM_2.5_) concentrations in 2019. PM_coarse_ is not represented as this measure was calculated separately

Residential levels of nitrogen dioxide (NO_2_), particulate matter < 10 μm (PM_10_) and < 2.5 μm (PM_2.5_) were modelled for the entire cohort (Fig. 1). However, some parts of Sweden (mostly Northern Sweden) included areas not covered by the air pollution data due to different projections used for the air pollution models and the residential mapping. This resulted in lack of air pollution data for a small number of the 1 000-m grid cells (68 out of 105 099 cells). The air pollution models, developed by the Swedish Environmental Research Institute, have been described by Gustafsson et al. [18]. Gridded concentrations for NO_2_, PM_10_, and PM_2.5_ were calculated using a combination of regional and urban background data at a 1 × 1 km resolution. The regional background concentrations were derived by interpolating data from national and regional air quality monitoring stations, taking seasonal variations into account through bi-monthly averaging. The urban background was modelled with the URBAN model, which adjusted concentrations based on population density and local sources, such as traffic, within each urban grid. This model also incorporated factors such as local meteorology (mixing height and wind speed), traffic patterns, and population distribution patterns to refine urban estimates. Air pollution concentrations were from 2019, and in our analyses we assume that the constant values are representative of exposure levels from 1991 to 2015. Using the modelled levels of particulate matter, we also calculated the concentration of PM_coarse_ by taking the difference between PM_10_ and PM_2.5_. This was done to specifically capture particles of sizes < 10 μm and ≥ 2.5 μm.

Individual air pollution exposure was based on the participants’ residence at the different time points. During pregnancy, the yearly average exposure levels were used for participants who resided in the same place throughout the entire pregnancy. For participants who moved during pregnancy, exposure levels were calculated as a weighted average, based on the proportion of the pregnancy spent at each residence and the corresponding yearly average exposure at each place. At 35 years of age and 2 years after the last delivery, the yearly average exposure levels were used.

## Statistical analysis

To estimate study population characteristics, we calculated mean and standard deviation for continuous variables and frequencies and percentages for categorical variables. Correlations between exposures were calculated using Pearson correlations.

Associations between air pollution and breast cancer were evaluated using Cox proportional hazards regression to estimate hazard ratios (HRs) and 95% confidence intervals (CIs), using time-on-study as time scale in order to ensure the same follow up time from exposure to outcome/censoring in all analyses. Individuals were followed from their first delivery until breast cancer diagnosis, with censoring for turning 50 years, 20 years since last delivery, emigration from Sweden, death, or end of follow up (December 31st, 2020). The proportional hazards assumption was assessed by visual inspection of Schoenfield residuals.

Since our primary focus was air pollution exposure during pregnancy, we first estimated associations in two main models using average residential exposure during 1) the first pregnancy, and 2) the last pregnancy before censoring, where the start of follow up was the first and last delivery, respectively. To assess the impact of timing of the exposure, we thereafter estimated associations for exposure levels at 35 years of age (regardless of pregnancy status), and at a time point outside of pregnancy, 2 years after the last delivery. To further compare the time points, we estimated HRs within participants with complete exposure data at all four time points.

Each exposure (NO_2_, PM_10_, PM_2.5,_ and PM_coarse_) was modelled separately and analyzed both as continuous variables (NO_2_ and PM_10_ per 10 μg/m^3^ increase, and PM_2.5_ and PM_coarse_ per 5 μg/m^3^ increase), and categorical variables, divided by quartiles. To capture possible non-linear effects, the exposures were modelled using restricted cubic spline functions with 4 knots (at quantiles 0.05, 0.35, 0.65, and 0.95). ANOVA tests were used to compare the fits between the linear model and the model with the spline.

Several subgroup analyses were conducted. To account for differences in tumor invasiveness, we estimated separate HRs for invasive tumors and DCIS, censoring participants with other tumor types at diagnosis. For invasive breast cancer cases with available information, we considered the impact of family history of breast and/or ovarian cancer among first-degree relatives by adding it as an additional confounder and also stratified the analyses by family history (yes, no). Potential interaction between the air pollutants and: 1) smoking (smoker, non-smoker), 2) family history of breast and/or ovarian cancer (yes, no), and 3) education level (primary school up to 9 years, secondary school, and postsecondary school) was assessed by including interaction terms in models with exposure levels during the first pregnancy. Furthermore, we applied a nested case–control (NCC) design, including invasive cases in the Swedish National Quality Register for Breast Cancer (NKBC) and five time-matched controls per case (additionally matched on birth year and age at first delivery), to estimate HRs stratified by breast cancer subtype (ER/PR-positive breast cancer, HER2-positive breast cancer, and triple-negative breast cancer) and by whether cases were screening-detected or not. For stratified analyses of screening and non-screening-detected cases we also conducted analyses limiting cases and controls to women between 40–49 years of age (to represent women eligible for the Swedish national screening program). In all analyses within the NCC design, we included only cases either classified as premenopausal in the register (defined as < 6 months since last menstruation) or diagnosed < 45 years of age to reduce the possibility of misclassification of menopausal status. Average air pollution levels during the last pregnancy were used as the exposures. The stratified Cox regression model (stratified on case–control set) was used to estimate the HRs.

Finally, we performed four sensitivity analyses: 1) using age as time scale in the main models with exposure levels at first pregnancy, last pregnancy, and 2 years after the last delivery, 2) censoring pregnancy-associated breast cancer (defined as cancer during pregnancy or within 1 year after delivery), 3) censoring breast cancer cases diagnosed within one year after start of follow up, and 4) stratifying analyses by time period. In the stratified analyses, we created three subsets of the study population based on the years of first and last delivery (1991–1995, 1996–2000, and 2001–2005). In all groups, end of follow up were set to 15 years after the last delivery. We included invasive cases and estimated associations for NO_2_ and PM_10_ exposure levels during the first and last pregnancy.

The choice of potential confounders was based on directed acyclic graphs (DAGs) and all models included age at first delivery, calendar year, country of birth, smoking, education level, marital status and the weighted Charlson comorbidity index. For analyses of exposures during the last pregnancy, at 35 years of age, and 2 years after the last delivery, we further adjusted for parity. Missing values were addressed so that if the missing was $\ge$ 0.1%, it was grouped in the category “unknown”, and if it was < 0.1%, participants with missing data were excluded from analyses.

All statistical analyses were carried out in R (v. 4.2.1) and R Studio (v. 2022.7.2.576) [19]. P-values of < 0.05 were considered significant.

## Results

At first delivery, the cohort included 1 019 076 women, with a median follow-up time of 14.9 years. During the study period, 12 085 participants (1.2%) were diagnosed with breast cancer (10 497 with invasive tumors). For all participants, the mean (SD) age at first delivery was 27.9 (4.9) years and the mean (SD) age at last delivery was 31.7 (4.8) years. Women who developed breast cancer were, on average, older at the first and last pregnancy (Table 1). Between the start of the first and last pregnancy, 44.8% of participants moved at least once, and between the first pregnancy and two years after the last delivery, 65.2% moved at least once (Figure S2). Average concentrations of all exposures were greater during the first pregnancy compared to the last pregnancy (Table S1). Correlations between the air pollutants were high for all time points (range 0.72–0.98 at first pregnancy) (Table S2). Among the 8206 cases included in the NKBC, 281 (3.4%) were classified as postmenopausal (Table S3).

**Table 1:** Participant characteristics

Variable	All participants (N = 1 019 076)	All breast cancer cases (N = 12 085)	Invasive breast cancer cases (N = 10 497)	DCIS cases (N = 1 393)
Age at diagnosis, *mean (SD)*	N/A	41.8 (5.1)	41.7 (5.1)	42.5 (4.6)
Age at first delivery, *mean (SD)*^a^	27.9 (4.9)	29.5 (4.6)	29.5 (4.6)	29.9 (4.6)
Age at last delivery, *mean (SD)*	31.7 (4.8)	32.8 (4.2)	32.8 (4.2)	33.2 (4.3)
*Total number of deliveries, n (%)*				
1	209 696 (20.6%)	2 824 (23.4%)	2 475 (23.6%)	316 (22.7%)
2	570 716 (56.0%)	6 900 (57.1%)	5 985 (57.0%)	794 (57.0%)
3	199 870 (19.6%)	2 036 (16.8%)	1 758 (16.7%)	245 (17.6%)
4	38 794 (3.8%)	325 (2.7%)	279 (2.7%)	38 (2.7%)
*Education, n (%)* ^*b*^				
Primary school up to 9 years	98 077 (9.6%)	816 (6.8%)	727 (6.9%)	80 (5.7%)
Secondary school	450 686 (44.2%)	5 288 (43.8%)	4 597 (43.8%)	612 (43.9%)
Postsecondary school	445 833 (43.7%)	5 779 (47.8%)	4 995 (47.6%)	679 (48.7%)
Unknown	24 480 (2.4%)	202 (1.7%)	178 (1.7%)	22 (1.6%)
*Civil status, n (%)* ^*a, b*^				
Married/registered partnership	376 394 (36.9%)	4 856 (40.2%)	4 227 (40.3%)	554 (39.8%)
Unmarried	623 210 (61.2%)	6 987 (57.8%)	6 059 (57.7%)	811 (58.2%)
Divorced	18 902 (1.9%)	237 (2.0%)	206 (2.0%)	28 (2.0%)
Widow	446 (0.0%)	5 (0.0%)	5 (0.0%)	0 (0.0%)
*CCIw (dichotomous), n (%)* ^*b*^				
No reported comorbidities	987 328 (96.9%)	11 862 (98.2%)	10 318 (98.3%)	1 357 (97.4%)
Any comorbidity (CCIw 1–7)	31 748 (3.1%)	223 (1.8%)	179 (1.7%)	36 (2.6%)
*Smoking, n (%)* ^*b*^				
Non-smoker	868 255 (85.2%)	10 213 (84.5%)	8 845 (84.3%)	1 198 (86.0%)
Smoker	98 745 (9.7%)	1 204 (10.0%)	1 072 (10.2%)	118 (8.5%)
Unknown	52 076 (5.1%)	668 (5.5%)	580 (5.5%)	77 (5.5%)
*Country of birth, n (%)*				
Sweden	838 102 (82.2%)	10 305 (85.3%)	8 951 (85.3%)	1 186 (85.1%)
Rest of Europe	78 391 (7.7%)	793 (6.6%)	703 (6.7%)	79 (5.7%)
Non-European country/Unknown	102 583 (10.1%)	987 (8.2%)	843 (8.0%)	128 (9.2%)
*Family history of breast/ovarian cancer, n (%)*				
Yes	76 971 (7.6%)	1 928 (16.0%)	1 674 (15.9%)	221 (15.9%)
No	664 232 (65.2%)	6 989 (57.8%)	6 087 (58.0%)	788 (56.6%)
Unknown	277 873 (27.3%)	3 168 (26.2%)	2 736 (26.1%)	384 (27.6%)

CCIw, weighted Charlson comorbidity index; DCIS, Ductal Carcinoma In Situ; SD, Standard deviation

^a^Includes < 0.1% missing

^b^Measured at first delivery

**Fig. 2:**
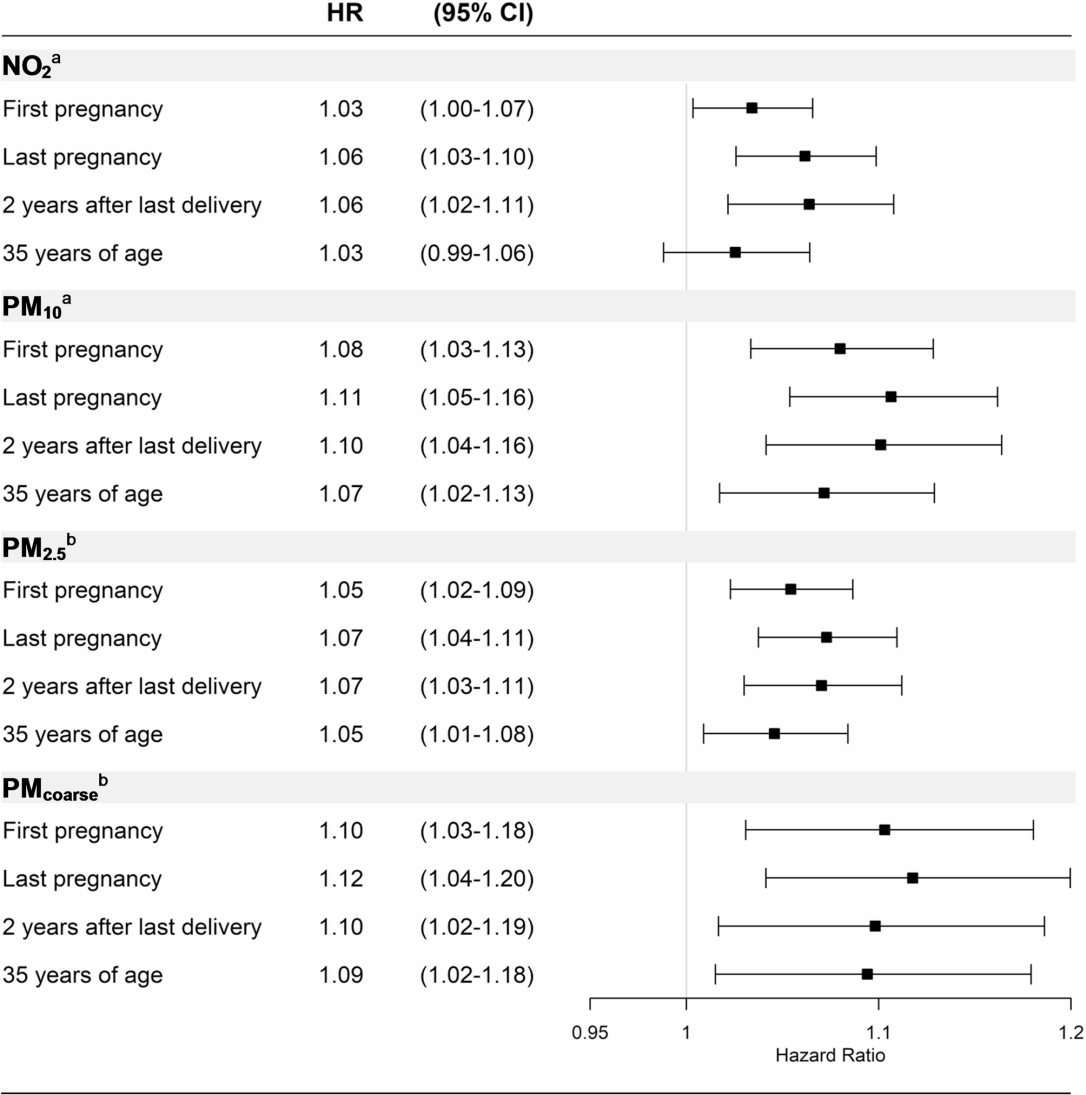
Associations between the air pollution exposures modelled as continuous variables and breast cancer, by time period. ^a^Per 10 µg/m^3^ increase. ^b^Per 5 µg/m^3^ increase. Models adjusted for age at first delivery, calendar year, country of birth, smoking, socioeconomic status, and Charlson comorbidity index. Last pregnancy, 2 years after last delivery, and 35 years of age additionally adjusted for total number of deliveries. HR: Hazard Ratios, CI: Confidence intervals

As results from the restricted cubic spline analyses and ANOVA tests indicated a linear relationship between exposures and breast cancer risk, except for PM_10_ and PM_2.5_ during the first pregnancy (Figure S3 A–D), we proceeded to primarily focus on the continuous models. In these analyses, all exposures were positively associated with breast cancer risk, regardless of exposure timepoint. For particulate matter, highest estimates were observed for exposures during the last pregnancy, HR_PM10_ = 1.11 (95% CI = 1.05, 1.16) per 10 μg/m^3^, HR_PM2.5_ = 1.07 (95% CI = 1.04, 1.11) per 5 μg/m^3^, and HR_PMcoarse_ = 1.12 (95% CI = 1.04, 1.20) per 5 μg/m^3^. For NO_2_, the highest estimate was observed for exposure at 2 years after the last delivery, HR_NO2_ = 1.06 (95% CI = 1.02, 1.11) per 10 μg/m^3^. Estimates for exposure levels at 35 years of age were similar to the first pregnancy, but slightly attenuated (Fig. 2). The HRs were similar when using age as the time scale (Table S4). In quartile analyses the differences between time periods were less pronounced, but the trend of positive associations between all exposures and breast cancer risk remained (Figure S4). In analyses utilizing the same population at all time points, differences between estimates at first pregnancy and at 35 years of age where less pronounced and HRs were highest for exposure levels 2 years after the last delivery (Figure S5). We found no violations of the proportional hazards assumption.

**Fig. 3:**
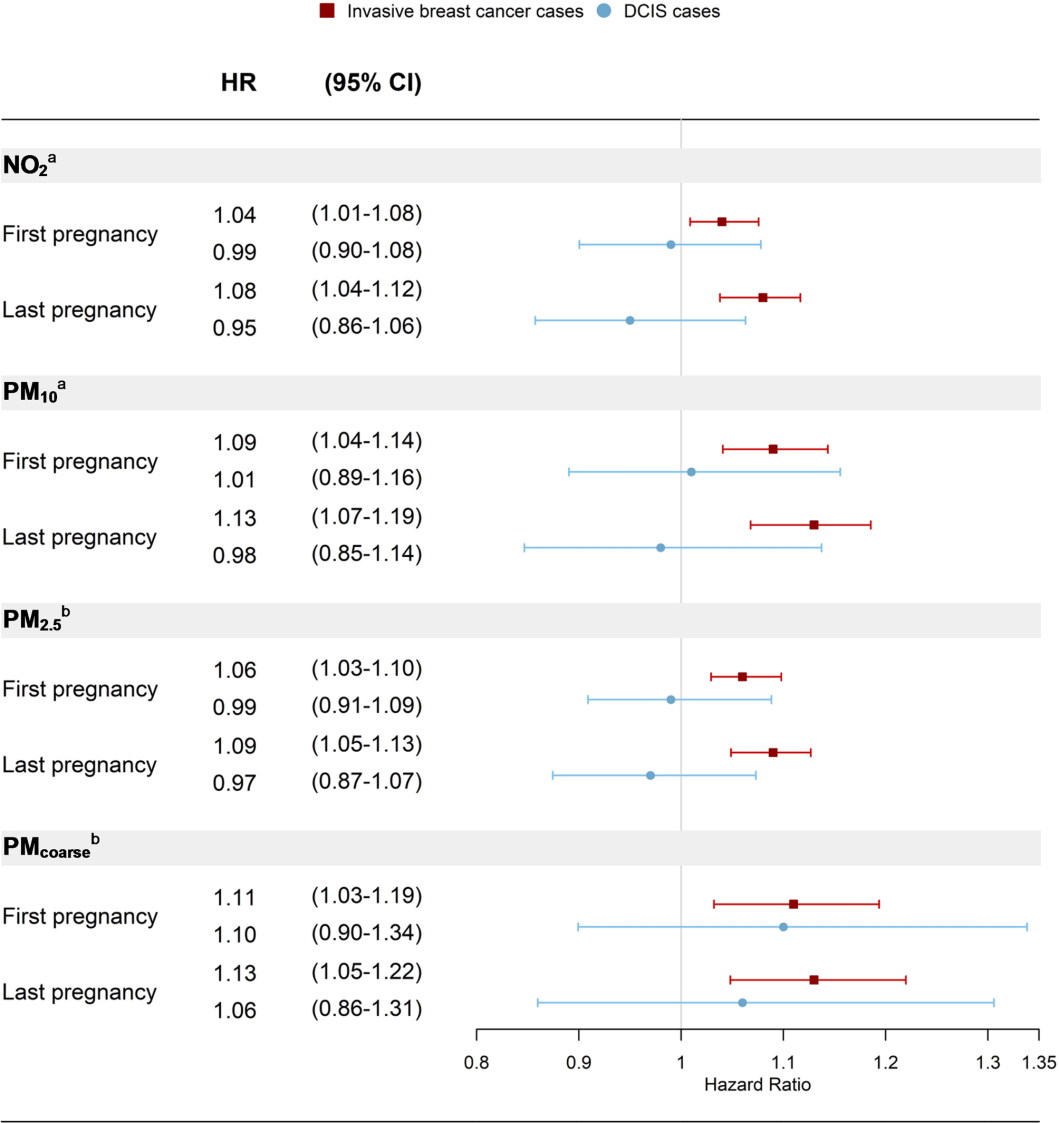
Associations between the air pollution exposures and breast cancer, by pregnancy period and tumor invasiveness. ^a^Per 10 µg/m^3^ increase. ^b^Per 5 µg/m^3^ increase. Models adjusted for age at first delivery, calendar year, country of birth, smoking, socioeconomic status, and Charlson comorbidity index. Last pregnancy additionally adjusted for total number of deliveries. DCIS: Ductal Carcinoma In Situ, HR: Hazard Ratios, CI: Confidence intervals

When we assessed associations separately in invasive and DCIS cases, the positive association remained for invasive breast cancer only and all associations for DCIS cases were close to null (Fig. 3). Adjusting for family history of breast and/or ovarian cancer attenuated the estimates slightly for first pregnancy exposures but did not impact associations for last pregnancy exposures (Table S5). Stratified analyses showed no clear difference by family history (Table S6). We did not identify any significant interactions between the air pollutants and family history, smoking, or education level. The stratum-specific effects of the air pollutants are shown in Table S7. In analyses of tumor subtypes, the HRs were positive for all air pollutants regardless of tumor subtype, reaching significance for NO_2_ and PM_2.5_ in ER/PR-positive breast cancer (Table 2). When evaluating associations by whether tumors were screening-detected or not, we found statistically significant positive associations between all air pollutants and non-screening-detected breast cancer and positive but non-significant associations for screening-detected cases (Table 3). Analyses of invasive cases and non-screening-detected cases of all ages were similar to those restricted to 40–49 years of age at diagnosis (Table S8).

**Table 2:** Associations between mean exposures during the last pregnancy period and premenopausal breast cancer, by tumor subtype

	ER/PR + n = (4748 cases/23 740 controls)	HER2 + n = (1401 cases/7005 controls)	Triple-negative n = (920 cases/4600 controls)
*NO*_*2*_			
HR (95% CI)^a^	1.08 (1.02–1.15)	1.11 (0.99–1.25)	1.08 (0.94–1.24)
*PM*_*10*_			
HR (95% CI)^a^	1.08 (0.99–1.18)	1.09 (0.93–1.28)	1.10 (0.90–1.34)
*PM*_*2.5*_			
HR (95% CI)^b^	1.06 (1.00–1.13)	1.08 (0.97–1.21)	1.04 (0.90–1.19)
*PM*_*coarse*_			
HR (95% CI)^b^	1.06 (0.93–1.20)	1.02 (0.81–1.29)	1.27 (0.95–1.69)

Models adjusted for calendar year, country of birth, smoking, socioeconomic status, Charlson comorbidity index, and total number of deliveries

HR, Hazard Ratios; CI, Confidence intervals

^a^Per 10 µg/m^3^ increase

^b^Per 5 µg/m^3^ increase

**Table 3:** Associations between mean exposures during the last pregnancy period and premenopausal breast cancer, by detection method

	All invasive cases 40–49 years of age^a^ n = (5661 cases/28 305 controls)	Screening-detected 40–49 years of age^a^ n = (2382 cases/11 910 controls)	Non-screening-detected 40–49 years of age^a^ n = (3256 cases/16 280 controls)
*NO*_*2*_			
HR (95% CI)^b^	1.09 (1.03–1.15)	1.02 (0.93–1.12)	1.12 (1.05–1.21)
*PM*_*10*_			
HR (95% CI)^b^	1.11 (1.03–1.21)	1.08 (0.95–1.23)	1.13 (1.02–1.26)
*PM*_*2.5*_			
HR (95% CI)^c^	1.08 (1.02–1.14)	1.07 (0.99–1.17)	1.08 (1.01–1.16)
*PM*_*coarse*_			
HR (95% CI)^c^	1.12 (1.00–1.26)	1.01 (0.84–1.21)	1.20 (1.03–1.40)

Models adjusted for calendar year, country of birth, smoking, socioeconomic status, Charlson comorbidity index, and total number of deliveries

HR, hazard ratios; CI, confidence intervals

^a^Includes cases diagnosed the year they turned 40

^b^Per 10 µg/m^3^ increase

^c^Per 5 µg/m^3^ increase

Sensitivity analyses excluding pregnancy-associated breast cancer and cases diagnosed within one year after start of follow up yielded HRs similar to the main models (Table S9 and Table S10). In analyses stratified by time periods for the first and last delivery, HRs were lowest in the earliest time period (1991–1995) for both NO_2_ and PM_10_. For NO_2_, we observed highest HRs for the latest time period (2001–2005). For PM_10_, HRs were similar between the time periods 1996–2000 and 2001–2005. Across all time periods, HRs were higher in the last pregnancy compared to the first pregnancy (Table S11).

## Discussion

In this population-based analysis including more than 12 000 cases of breast cancer, we found that exposure to ambient air pollution (NO_2_, PM_10_, PM_2.5,_ and PM_coarse_) was consistently associated with an increased risk of invasive early-onset breast cancer, across the different time periods. We observed statistically significant positive associations for exposures during the first and last pregnancy, 2 years after the last delivery, as well as for exposure levels at 35 years of age, with the exception of NO_2_.

Although, to the best of our knowledge, no previous studies have investigated the association between air pollution exposure during pregnancy and premenopausal breast cancer, some studies have focused on other sensitive time periods. For example, a US based cohort that evaluated air pollution during childhood found inconsistent associations with breast cancer risk [20] and another study identified a positive association between traffic emissions during menarche and premenopausal breast cancer among non-smokers [21]. Regarding exposures during pregnancy, a recent study investigated ambient aromatic hydrocarbons (PAH) exposure in relation to breast tissue composition in mothers and daughters and found no associations [22]. In our study we observed somewhat stronger associations for exposures during the last pregnancy period compared to the first pregnancy period, both in the main models and in analyses within the same population. This was somewhat contrary to our initial hypothesis as we had expected stronger effects for the first pregnancy (representing the first full differentiation of the mammary glands [23]), one potential reason for this could be that the last pregnancy occurs closer in time to the breast cancer diagnosis, but the attenuated associations observed in women at 35 years of age undermines this. Interestingly, however, associations for exposures evaluated 2 years after the last delivery were consistently stronger than those at 35 years of age and very similar to those for exposures evaluated at the last pregnancy. This might reflect that the pregnancy window of susceptibility extends for at least two years after delivery and includes both the lactation period and later tissue dedifferentiation.

Contrary to many previous studies of air pollution and breast cancer risk we found higher risk estimates for associations with PM compared to NO_2_. This could be related to the fact that PM-measures may differ in both composition and effect, depending on the exposure source, and our models might capture particles with different properties than studies conducted in other countries. Particles are also often described as having endocrine disrupting properties [24], with possible implications for receptor positive breast cancer, and a recent study reported positive associations between PM_2.5_ and ER-positive, but not ER-negative breast cancer [25]. Our findings, however, primarily indicated an increased risk regardless of tumor subtype, but with statistically significant positive associations only for NO_2_ and PM_2.5_ in ER/PR positive breast cancer.

The main strengths of the study were the large population-based sample and large number of early-onset breast cancer cases, allowing us to evaluate air pollution exposure in women younger than 50 years of age [26]. We also had information about tumor invasiveness and, for a subset of the cases, tumor subtype and whether the cancer was screening-detected or not, making it possible to analyze these characteristics separately. Furthermore, we had access to residential addresses during the entire study period, including moving dates, which the air pollutants could been linked to. We also had access to many important breast cancer risk factors, including age at first delivery and family history of breast and/or ovarian cancer.

A major limitation in our study is the possibility of exposure misclassification and loss of contrast between exposure periods. This is related to our air pollution model, which was based on data from 2019 and assumed constant levels during the study period despite the fact that air pollution levels in Sweden have generally decreased since the 1990s [18]. Furthermore, the spatial resolution of the exposure model (1 km2) may not fully capture within-city variations, which is a further cause of exposure misclassification, and may suggest that the associations observed in the present study are underestimated. However, despite the fact that relying on exposure models for a single year is an important limitation, spatial contrasts in air pollution tend to remain relatively stable over time and we were able to completely account for residential mobility in all our analyses. In a separate cohort, we also assessed the correlation of PM_2.5_ and NOₓ concentrations between 1990, 2000, and 2010 in a subset of the study area (Malmö) [27]. The correlation coefficients were all above 0.7 (for years ten years apart), supporting the assumption that spatial contrasts are stable over time and lending robustness to our exposure assessment. Therefore, over the 24-year follow-up in this study, although a subset of participants may have experienced substantial changes in local infrastructure, the spatial contrasts in air pollution for the majority of participants are likely to have remained relatively stable, as exemplified by data from Malmö [27]. Detailed data on temporal changes in spatial contrasts for the entirety of Sweden are unfortunately not available; however, it is expected that spatial contrasts remain high on a population-level. In sensitivity analyses stratifying between different time periods we did identify differences in the magnitude of estimates, observing higher estimates for women exposed during the later compared to earlier calendar years. These differences could indicate increased exposure misclassification in earlier years due to altered air pollution levels over time, but it is also possible that they reflect altered screening and diagnostic practices between the early 1990s and 2000s. Furthermore, we consistently observed higher HR estimates for the last pregnancy compared to the first pregnancy (regardless of calendar year of exposure). This is in line with the results in the main analyses, making it unlikely that these differences are due to exposure misclassification alone.

Another limitation was lack of data on menopausal status at diagnosis, which was only available for women diagnosed after 2007. We therefore used breast cancer diagnosed before age 50 as a proxy for premenopausal breast cancer. This may have led to some misclassification of menopausal status, which could have affected our outcome estimates. However, in the subset of cases in the NKBC, only 3.4% were classified as postmenopausal at diagnosis. Finally, we lacked information about some potentially important covariates, such as alcohol consumption, physical activity and body mass index (BMI). BMI is inversely associated with premenopausal breast cancer [28–30], but is not thought to be causally associated with air pollution, and is therefore unlikely to have confounded our associations.

## Conclusions

Taken together, our findings suggest that exposure to ambient air pollution, both NO_2_ and different PM sizes, is associated with an increased risk of invasive early-onset breast cancer. This could have implications for future cancer prevention efforts, for example by justifying directing preventative efforts towards groups that could benefit more from interventions. We found some indications of stronger associations during sensitive times (especially during the last pregnancy and two years after) compared to exposure at a set age, but those differences would have to be confirmed in later studies, ideally using more detailed air pollution data, for example monthly instead of yearly averages. This would be of high future interest in order to evaluate exposure not only during the entire pregnancy, but also during the separate trimesters (and post-partum), as these are all connected to different stages of breast tissue differentiation [23].

## Supplementary Information

Below is the link to the electronic supplementary material.

Supplementary Material 1

## Data Availability

The data that support the findings of this study are available from the Swedish National Board of Health and Welfare and the Swedish National Quality Register for Breast Cancer, subject to obtaining the necessary ethical approvals. Air pollution data was modelled by the Swedish Environmental Research Institute (IVL), and additional restrictions may apply to the availability of this data. Further information is available from the corresponding authors upon request.

## Abbreviations

DAGs: Directed acyclic graphs
DCIS: Ductal carcinoma in situ
ER: Estrogen receptor
NCC: Nested case–control
NKBC: Swedish National Quality Register for Breast Cancer
NO_2_: Nitrogen dioxide
PM: Particulate matter
PR: Progesterone receptor
wCCI: Weighted Charlson comorbidity index
