# Supplementary material for: Exposure to ambient air pollution during pregnancy and risk of early-onset breast cancer
Source: Breast Cancer Res. 2025 Nov 13;27:203. doi: 10.1186/s13058-025-02165-9 (PMC12616941; doi:10.1186/s13058-025-02165-9)
Supplement: Supplementary file 1 — Supplementary Material 1 [file 13058_2025_2165_MOESM1_ESM.docx]

**Supplemental Material**

**Exposure to ambient air pollution during pregnancy and risk of early-onset breast cancer**

Jessica Edlund^1^, Wendy Yi-Ying Wu^1^, Malin Gustafsson^2^, Jenny Lindén^2^, Anna Oudin^3, 4^, Sophia Harlid^1^

*^1^Department of Diagnostics and Intervention, Oncology, Umeå University, Umeå 901 87, Sweden*

*^2^IVL Swedish Environmental Research Institute, Gothenburg, Sweden*

*^3^Department of Public Health and Clinical Medicine, Division for Sustainable Health, Umeå University, Umeå, Sweden*

*^4^Division of Occupational and Environmental Medicine, Department of Laboratory Medicine, Lund University, Lund, Sweden*

**Table of Contents: Page**

Figure S1 (Flow chart of study population selection) 1

Figure S2 (Residential moves during the study period) 2

Figure S3 (Restricted cubic spline analyses, A, B, C, and D) 3

Figure S4 (Models with exposures divided by quartiles) 7

Figure S5 (Comparison of time points within the same population) 8

Table S1 (Exposure levels at different time points) 9

Table S2 (Exposure correlations) 10

Table S3 (Participant characteristics for nested case-control data set) 11

Table S4 (Models first pregnancy, age as time scale) 13

Table S5 (Models adjusted for family history) 14

Table S6 (Stratified analyses by family history) 15

Table S7 (Interaction analyses with smoking, family history, and education) 16

Table S8 (Stratified analyses by detection method, including all ages at diagnosis) 17

Table S9 (Models without pregnancy-associated breast cancer) 18

Table S10 (Models without cases diagnosed within 1 year after start of follow-up) 19

Table S11 (Stratified analyses by time period) 20

**
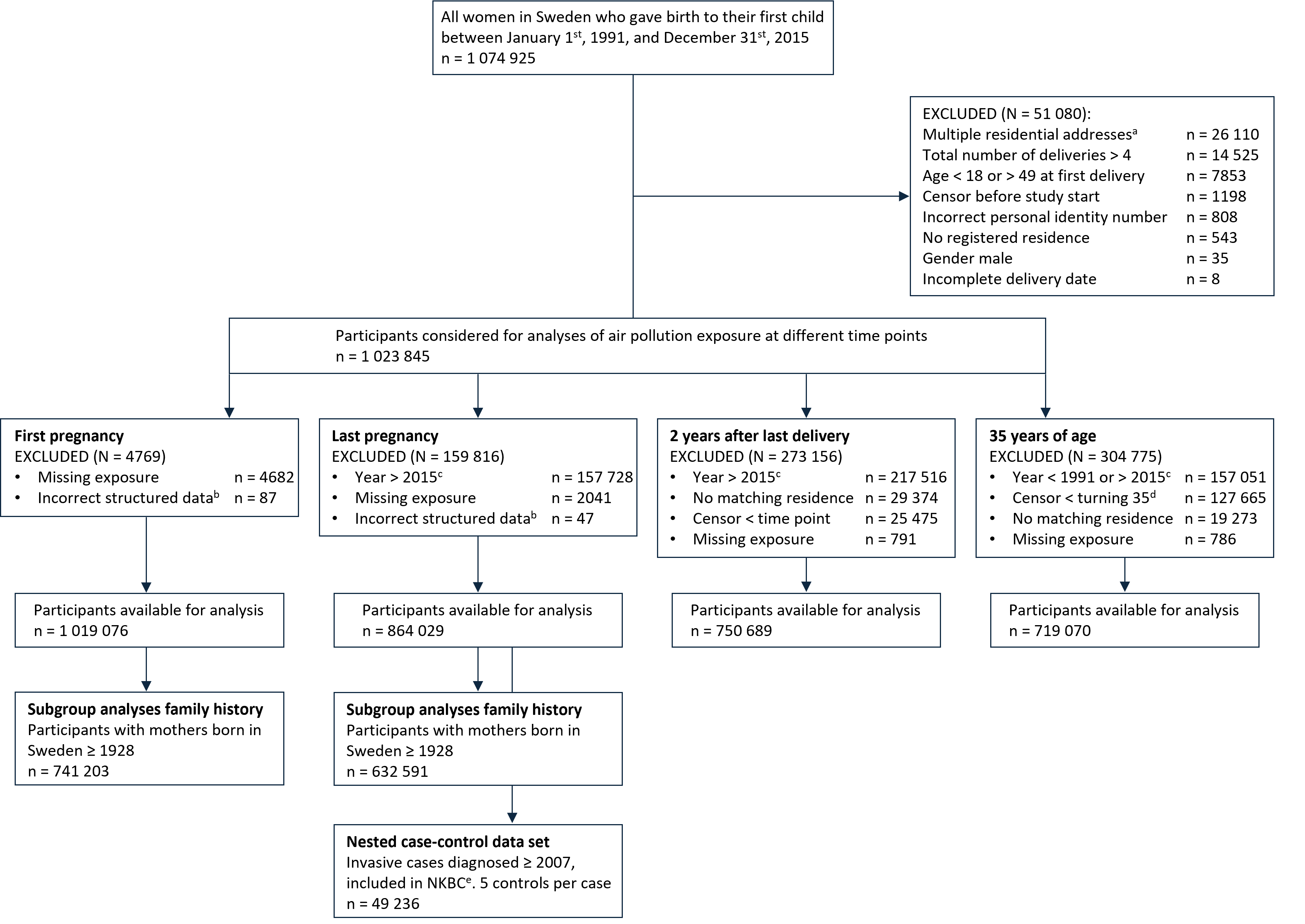
**

**Figure S1.** Flow chart of study population selection.

^a^Multiple residential addresses: Different addresses for the same time period.

^b^Incorrect structured data: Overlapping pregnancy periods (a new pregnancy started before or on the same date as the delivery of the previous pregnancy).

^c^Year < 1991 or > 2015: These years fall outside the study period. Years after 2015 were specifically excluded to allow for at least five years of follow-up.

^d^Censor < turning 35: Breast cancer diagnosis, emigration from Sweden, death, or end of follow up (December 31^st^, 2020) before 35 years of age.

^e^NKBC: Swedish National Quality Register for Breast Cancer.

**Figure S2.** Residential moves during the study period. Moves are defined as a change in residential NO₂ exposure levels.

^a^N (%) of individuals who moved between the time points of interest. Percentages are calculated among individuals with exposure data at both time points.

^b^Number of individuals with exposure data at that time point.

**Figure S3**. Restricted cubic spline analyses with 4 knots (at quantiles 0.05, 0.35, 0.65, and 0.95), plotted using the minimum value as reference. P-values correspond to ANOVA tests, where we compared the fits between the linear model and the model with the spline. A) Yearly average exposures during the first pregnancy, B) Yearly average exposures during the last pregnancy, C) Yearly average exposures at 2 years after last delivery, D) Yearly average exposures at 35 years of age.

HR: Hazard Ratios, NO_2_: nitrogen dioxide, PM: particulate matter

**Figure S4.** Associations between the air pollution exposures modelled as categorical variables (divided by quartiles) and breast cancer, by time period.

**First pregnancy quartile cut points:**
NO_2_: Q1: <3.5 μg/m^3^; Q2: 3.5 μg/m^3^ to <5.7 μg/m^3^; Q3: 5.7 μg/m^3^ to <8.4 μg/m^3^; Q4: >8.4 μg/m^3^.
PM_10_: Q1: <9.0 μg/m^3^; Q2: 9.0 μg/m^3^ to <11.0 μg/m^3^; Q3: 11.0 μg/m^3^ to <13.2 μg/m^3^; Q4: >13.2 μg/m^3^.
PM_2.5_: Q1: <5.8 μg/m^3^; Q2: 5.8 μg/m^3^ to <7.3 μg/m^3^; Q3: 7.3 μg/m^3^ to <8.9 μg/m^3^; Q4: >8.9 μg/m^3^.
PM_coarse_: Q1: <3.0 μg/m^3^; Q2: 3.0 μg/m^3^ to <3.7 μg/m^3^; Q3: 3.7 μg/m^3^ to <4.6 μg/m^3^; Q4: >4.6 μg/m^3^.
**Last pregnancy quartile cut points:**
NO_2_: Q1: <2.9 μg/m^3^; Q2: 2.9 μg/m^3^ to <5.0 μg/m^3^; Q3: 5.0 μg/m^3^ to <7.5 μg/m^3^; Q4: >7.5 μg/m^3^.
PM_10_: Q1: <8.6 μg/m^3^; Q2: 8.6 μg/m^3^ to <10.7 μg/m^3^; Q3: 10.7 μg/m^3^ to <12.7 μg/m^3^; Q4: >12.7 μg/m^3^.
PM_2.5_: Q1: <5.4 μg/m^3^; Q2: 5.4 μg/m^3^ to <7.0 μg/m^3^; Q3: 7.0 μg/m^3^ to <8.4 μg/m^3^; Q4: >8.4 μg/m^3^.
PM_coarse_: Q1: <2.9 μg/m^3^; Q2: 2.9 μg/m^3^ to <3.6 μg/m^3^; Q3: 3.6 μg/m^3^ to <4.5 μg/m^3^; Q4: >4.5 μg/m^3^.
**2 years after last delivery quartile cut points:**
NO_2_: Q1: <2.8 μg/m^3^; Q2: 2.8 μg/m^3^ to <4.6 μg/m^3^; Q3: 4.6 μg/m^3^ to <6.9 μg/m^3^; Q4: >6.9 μg/m^3^.
PM_10_: Q1: <8.4 μg/m^3^; Q2: 8.4 μg/m^3^ to <10.5 μg/m^3^; Q3: 10.5 μg/m^3^ to <12.3 μg/m^3^; Q4: >12.3 μg/m^3^.
PM_2.5_: Q1: <5.3 μg/m^3^; Q2: 5.3 μg/m^3^ to <6.8 μg/m^3^; Q3: 6.8 μg/m^3^ to <8.1 μg/m^3^; Q4: >8.1 μg/m^3^.
PM_coarse_: Q1: <2.9 μg/m^3^; Q2: 2.9 μg/m^3^ to <3.6 μg/m^3^; Q3: 3.6 μg/m^3^ to <4.5 μg/m^3^; Q4: >4.5 μg/m^3^.
**35 years of age quartile cut points:**
NO_2_: Q1: <2.8 μg/m^3^; Q2: 2.8 μg/m^3^ to <4.8 μg/m^3^; Q3: 4.8 μg/m^3^ to <7.4 μg/m^3^; Q4: >7.4 μg/m^3^.
PM_10_: Q1: <8.5 μg/m^3^; Q2: 8.5 μg/m^3^ to <10.6 μg/m^3^; Q3: 10.6 μg/m^3^ to <12.6 μg/m^3^; Q4: >12.6 μg/m^3^.
PM_2.5_: Q1: <5.3 μg/m^3^; Q2: 5.3 μg/m^3^ to <6.9 μg/m^3^; Q3: 6.9 μg/m^3^ to <8.3 μg/m^3^; Q4: >8.3 μg/m^3^.
PM_coarse_: Q1: <2.9 μg/m^3^; Q2: 2.9 μg/m^3^ to <3.6 μg/m^3^; Q3: 3.6 μg/m^3^ to <4.5 μg/m^3^; Q4: >4.5 μg/m^3^.

HR: Hazard Ratios, CI: Confidence intervals, NO2: nitrogen dioxide, PM: particulate matter

**Figure S5.** Comparison of four time points within the same population (N = 644 213 participants).

NO_2_ and PM_10_: Per 10 µg/m^3^ increase.

PM_2.5_ and PM_coarse_: Per 5 µg/m^3^ increase.

Models adjusted for age at first delivery, calendar year, country of birth, smoking, socioeconomic status, and Charlson comorbidity index. Last pregnancy, 2 years after last delivery, and 35 years of age additionally adjusted for total number of deliveries.

Of the participants, 170 737 (26.5 %) never moved. Moves are defined as a change in residential NO₂ exposure levels.

HR: Hazard Ratios, CI: Confidence intervals, NO_2_: nitrogen dioxide, PM: particulate matter

**Table S1.** Exposure levels [µg/m^3^] and follow-up time at different time points.

| **Variable** | **All participants** | **All breast cancer cases** | **Invasive breast cancer cases** | **DCIS cases** |
| --- | --- | --- | --- | --- |
| **Exposure first pregnancy** | **(N = 1 019 076)** | **(N = 12 085)** | **(N = 10 497)** | **(N = 1 393)** |
| NO_2_, *median (IQR)* | 5.7 (3.5, 8.4) | 5.9 (3.5, 8.8) | 5.9 (3.5, 8.8) | 5.7 (3.5, 8.5) |
| PM_10_, *median (IQR)* | 11.0 (9.0, 13.2) | 11.0 (9.1, 13.4) | 11.1 (9.1, 13.4) | 10.9 (9.0, 13.2) |
| PM_2.5_, *median (IQR)* | 7.3 (5.8, 8.9) | 7.4 (5.8, 9.0) | 7.4 (5.9, 9.0) | 7.2 (5.8, 8.9) |
| PM_coarse_*, median (IQR)* | 3.7 (3.0, 4.6) | 3.7 (3.0, 4.7) | 3.7 (3.0, 4.7) | 3.7 (3.1, 4.6) |
| Calendar year (first pregnancy), *median (range)* | 2004 (1991, 2015) | 1999 (1991, 2015) | 1999 (1991, 2015) | 2001 (1991, 2015) |
| Years of follow-up, *median (IQR)* | 14.9 (9.8, 20.0) | 12.3 (8.0, 16.6) | 12.3 (7.9, 16.6) | 12.7 (8.6, 16.6) |
|  |  |  |  |  |
| **Exposure last pregnancy** | **(N = 864 029)** | **(N = 11 878)** | **(N = 10 320)** | **(N = 1 364)** |
| NO_2_, *median (IQR)* | 5.0 (2.9, 7.5) | 5.0 (3.0, 7.8) | 5.1 (3.0, 7.8) | 4.7 (3.0, 7.3) |
| PM_10_, *median (IQR)* | 10.7 (8.6, 12.7) | 10.7 (8.7, 12.8) | 10.7 (8.7, 12.8) | 10.6 (8.6, 12.6) |
| PM_2.5_, *median (IQR)* | 7.0 (5.4, 8.4) | 7.1 (5.5, 8.5) | 7.1 (5.5, 8.5) | 6.9 (5.4, 8.2) |
| PM_coarse_*, median (IQR)* | 3.6 (2.9, 4.5) | 3.6 (3.0, 4.6) | 3.6 (3.0, 4.6) | 3.6 (3.0, 4.6) |
| Calendar year (last pregnancy), *median (range)* | 2006 (1991, 2015) | 2003 (1991, 2015) | 2003 (1991, 2015) | 2004 (1991, 2015) |
| Years of follow-up, *median (IQR)* | 12.5 (8.5, 16.9) | 8.9 (5.1, 13.0) | 8.9 (5.0, 12.9) | 9.3 (5.6, 13.0) |
|  |  |  |  |  |
| **Exposure 2 years after last delivery** | **(N = 750 689)** | **(N = 10 438)** | **(N = 9 035)** | **(N = 1 227)** |
| NO_2_, *median (IQR)* | 4.6 (2.8, 6.9) | 4.6 (2.8, 7.1) | 4.7 (2.8, 7.1) | 4.5 (2.8, 6.6) |
| PM_10_, *median (IQR)* | 10.5 (8.4, 12.3) | 10.5 (8.4, 12.5) | 10.6 (8.4, 12.5) | 10.4 (8.4, 12.1) |
| PM_2.5_, *median (IQR)* | 6.8 (5.3, 8.1) | 6.9 (5.3, 8.2) | 6.9 (5.3, 8.2) | 6.7 (5.3, 7.9) |
| PM_coarse_*, median (IQR)* | 3.6 (2.9, 4.5) | 3.6 (2.9, 4.5) | 3.6 (2.9, 4.5) | 3.6 (2.9, 4.5) |
| Calendar year (2 years after last delivery), *median (range)* | 2007 (1993, 2015) | 2005 (1993, 2015) | 2004 (1993, 2015) | 2005 (1993, 2015) |
| Years of follow-up, *median (IQR)* | 11.6 (8.1, 15.6) | 7.9 (4.5, 11.5) | 7.8 (4.5, 11.5) | 7.9 (4.7, 11.4) |
|  |  |  |  |  |
| **Exposure 35 years of age** | **(N = 719 070)**^a^ | **(N = 10 485)** | **(N = 9 043)** | **(N = 1 266)** |
| NO_2_, *median (IQR)* | 4.8 (2.8, 7.4) | 4.9 (2.9, 7.6) | 5.0 (3.0, 7.7) | 4.6 (2.8, 7.0) |
| PM_10_, *median (IQR)* | 10.6 (8.5, 12.6) | 10.7 (8.6, 12.7) | 10.7 (8.6, 12.8) | 10.5 (8.4, 12.3) |
| PM_2.5_, *median (IQR)* | 6.9 (5.3, 8.3) | 7.0 (5.4, 8.4) | 7.1 (5.4, 8.4) | 6.7 (5.3, 8.1) |
| PM_coarse_*, median (IQR)* | 3.6 (2.9, 4.5) | 3.7 (3.0, 4.6) | 3.7 (3.0, 4.6) | 3.6 (3.0, 4.5) |
| Calendar year at 35, *median (range)* | 2007 (1991, 2015) | 2004 (1991, 2015) | 2004 (1991, 2015) | 2005 (1991, 2015) |
| Years of follow-up, *median (IQR)* | 12.3 (8.8, 15.0) | 8.8 (5.4, 11.6) | 8.8 (5.3, 11.6) | 8.9 (6.0, 11.4) |
|  |  |  |  |  |
| ^a^61 665 (8.6%) of the women were in a pregnancy period. | | | | |
| NO_2_: nitrogen dioxide, PM: particulate matter, IQR: Interquartile Range | | | | |

**Table S2.** Pearson correlations between the air pollutants at different time points.

| **First pregnancy** | NO_2_ | PM_10_ | | PM_2.5_ | | PM_coarse_ | |  |
| --- | --- | --- | --- | --- | --- | --- | --- | --- |
| NO_2_ | 1 | |  | |  | |  | |
| PM_10_ | 0.869 | | 1 | |  | |  | |
| PM_2.5_ | 0.869 | | 0.976 | | 1 | |  | |
| PM_coarse_ | 0.720 | | 0.879 | | 0.754 | | 1 | |
|  |  | |  | |  | |  | |
| **Last pregnancy** | NO_2_ | | PM_10_ | | PM_2.5_ | | PM_coarse_ | |
| NO_2_ | 1 | |  | |  | |  | |
| PM_10_ | 0.844 | | 1 | |  | |  | |
| PM_2.5_ | 0.840 | | 0.967 | | 1 | |  | |
| PM_coarse_ | 0.667 | | 0.849 | | 0.685 | | 1 | |
|  |  | |  | |  | |  | |
| **2 years after last delivery** | NO_2_ | | PM_10_ | | PM_2.5_ | | PM_coarse_ | |
| NO_2_ | 1 | |  | |  | |  | |
| PM_10_ | 0.824 | | 1 | |  | |  | |
| PM_2.5_ | 0.817 | | 0.959 | | 1 | |  | |
| PM_coarse_ | 0.634 | | 0.832 | | 0.642 | | 1 | |
|  |  | |  | |  | |  | |
| **35 years of age** | NO_2_ | | PM_10_ | | PM_2.5_ | | PM_coarse_ | |
| NO_2_ | 1 | |  | |  | |  | |
| PM_10_ | 0.848 | | 1 | |  | |  | |
| PM_2.5_ | 0.846 | | 0.966 | | 1 | |  | |
| PM_coarse_ | 0.664 | | 0.846 | | 0.681 | | 1 | |
|  |  | |  | |  | |  | |
| NO_2_: nitrogen dioxide, PM: particulate matter | | | | | | | | |

**Table S3.** Participant characteristics for invasive cases included in the National Quality Register for Breast Cancer and matched controls.

| **Variable** | **Cases** (N = 8 206) | **Controls** (N = 41 030) |
| --- | --- | --- |
| Age at diagnosis, *mean (SD)* | 42.6 (4.7) | N/A |
| Tumor subtype, *n (%)*^a^ |  | N/A |
| ER/PR+ | 5 318 (64.7%) |  |
| HER2+ | 1 543 (18.8%) |  |
| Triple-negative | 988 (12.0%) |  |
| Unknown | 375 (4.6%) |  |
| Detection method, *n (%)* |  | N/A |
| Screening-detected < 40 years of age | 25 (0.3%) |  |
| Screening-detected 40-49 years of age^b^ | 2 763 (33.7%) |  |
| Non-screening-detected | 5 384 (65.6%) |  |
| Unknown | 34 (0.4%) |  |
| Menopausal status at diagnosis, *n (%)*^c^ |  | N/A |
| Premenopausal | 7 397 (90.1%) |  |
| Postmenopausal | 281 (3.4%) |  |
| Unknown | 528 (6.4%) |  |
| Age at first delivery, *mean (SD)* | 29.2 (4.5) | 29.2 (4.5) |
| Age at last delivery, *mean (SD)* | 32.7 (4.2) | 31.9 (4.2) |
| Total number of deliveries, *n (%)* |  |  |
| 1 | 1 766 (21.5%) | 11 870 (28.9%) |
| 2 | 4 734 (57.7%) | 23 807 (58.0%) |
| 3 | 1 473 (18.0%) | 4 792 (11.7%) |
| 4 | 233 (2.8%) | 561 (1.4%) |
| Education, *n (%)*^d^ |  |  |
| Primary school up to 9 years | 488 (5.9%) | 2 664 (6.5%) |
| Secondary school | 3 471 (42.3%) | 18 786 (45.8%) |
| Postsecondary school | 4 204 (51.2%) | 19 267 (47.0%) |
| Unknown | 43 (0.5%) | 313 (0.8%) |
| Civil status, *n (%)*^d^ |  |  |
| Married/registered partnership | 4 228 (51.5%) | 20 010 (48.8%) |
| Unmarried | 3 685 (44.9%) | 19 913 (48.5%) |
| Divorced | 288 (3.5%) | 1 080 (2.6%) |
| Widow | 5 (0.1%) | 27 (0.1%) |
| CCIw (dichotomous), *n (%)*^d^ |  |  |
| No reported comorbidities | 7 965 (97.1%) | 39 750 (96.9%) |
| Any comorbidity (CCIw 1-7) | 241 (2.9%) | 1 280 (3.1%) |
| Smoking, *n (%)*^d^ |  |  |
| Non-smoker | 7 160 (87.3%) | 35 594 (86.8%) |
| Smoker | 580 (7.1%) | 3 246 (7.9%) |
| Unknown | 466 (5.7%) | 2 190 (5.3%) |
| Country of birth, *n (%)* |  |  |
| Sweden | 6 991 (85.2%) | 35 364 (86.2%) |
| Rest of Europe | 532 (6.5%) | 2 628 (6.4%) |
| Non-European country/Unknown | 683 (8.3%) | 3 038 (7.4%) |
| Family history of breast/ovarian cancer, *n (%)* |  |  |
| Yes | 1 299 (15.8%) | 3 881 (9.5%) |
| No | 4 922 (60.0%) | 27 744 (67.6%) |
| Unknown | 1 985 (24.2%) | 9 405 (22.9%) |
| ^a^Numbers do not sum to total because bilateral breast cancers with different subtypes were counted in both groups. | | |
| ^b^Includes cases diagnosed the year they turned 40. | | |
| ^c^Cases < 45 years with unknown menopausal status were classified as premenopausal (n = 246, 3.0%). | | |
| ^d^Measured at last delivery. | | |
| CCIw: weighted Charlson comorbidity index, SD: Standard deviation | | |

**Table S4.** Associations between the air pollution exposures and breast cancer, measured at first pregnancy, last pregnancy, and 2 years after last delivery, using age as the time scale.

| **Exposure** | **Continuous variable** | **Quartile 1** | **Quartile 2** | **Quartile 3** | **Quartile 4** |
| --- | --- | --- | --- | --- | --- |
| **First pregnancy** |  |  |  |  |  |
| NO_2_ |  |  |  |  |  |
| HR (95% CI)^a^ | 1.04 (1.01-1.07) | 1.0 (ref.) | 1.00 (0.95-1.05) | 1.07 (1.02-1.12) | 1.06 (1.01-1.12) |
| PM_10_ |  |  |  |  |  |
| HR (95% CI)^a^ | 1.08 (1.04-1.13) | 1.0 (ref.) | 1.08 (1.03-1.14) | 1.06 (1.01-1.12) | 1.11 (1.05-1.17) |
| PM_2.5_ |  |  |  |  |  |
| HR (95% CI)^b^ | 1.06 (1.03-1.09) | 1.0 (ref.) | 1.04 (0.99-1.09) | 1.09 (1.03-1.14) | 1.09 (1.03-1.14) |
| PM_coarse_ |  |  |  |  |  |
| HR (95% CI)^b^ | 1.11 (1.03-1.18) | 1.0 (ref.) | 1.05 (1.00-1.11) | 1.03 (0.98-1.09) | 1.07 (1.02-1.13) |
|  |  |  |  |  |  |
| **Last pregnancy** |  |  |  |  |  |
| NO_2_ |  |  |  |  |  |
| HR (95% CI)^a^ | 1.06 (1.02-1.10) | 1.0 (ref.) | 1.03 (0.98-1.08) | 1.00 (0.94-1.05) | 1.09 (1.04-1.15) |
| PM_10_ |  |  |  |  |  |
| HR (95% CI)^a^ | 1.11 (1.05-1.16) | 1.0 (ref.) | 1.07 (1.02-1.13) | 1.05 (1.00-1.11) | 1.11 (1.05-1.16) |
| PM_2.5_ |  |  |  |  |  |
| HR (95% CI)^b^ | 1.08 (1.04-1.11) | 1.0 (ref.) | 1.04 (0.99-1.10) | 1.08 (1.02-1.13) | 1.10 (1.05-1.16) |
| PM_coarse_ |  |  |  |  |  |
| HR (95% CI)^b^ | 1.11 (1.03-1.19) | 1.0 (ref.) | 1.04 (0.98-1.09) | 1.04 (0.98-1.09) | 1.07 (1.01-1.12) |
|  |  |  |  |  |  |
| **2 years after last delivery** |  |  |  |  |  |
| NO_2_ |  |  |  |  |  |
| HR (95% CI)^a^ | 1.06 (1.01-1.10) | 1.0 (ref.) | 1.04 (0.99-1.10) | 1.01 (0.95-1.07) | 1.06 (1.00-1.12) |
| PM_10_ |  |  |  |  |  |
| HR (95% CI)^a^ | 1.10 (1.04-1.16) | 1.0 (ref.) | 1.01 (0.95-1.06) | 1.05 (0.99-1.10) | 1.07 (1.01-1.13) |
| PM_2.5_ |  |  |  |  |  |
| HR (95% CI)^b^ | 1.07 (1.03-1.11) | 1.0 (ref.) | 1.02 (0.97-1.08) | 1.05 (1.00-1.11) | 1.07 (1.02-1.13) |
| PM_coarse_ |  |  |  |  |  |
| HR (95% CI)^b^ | 1.09 (1.01-1.17) | 1.0 (ref.) | 1.00 (0.95-1.06) | 1.00 (0.95-1.06) | 1.05 (0.99-1.10) |
|  |  |  |  |  |  |
| ^a^Per 10 µg/m^3^ increase. | | | | | |
| ^b^Per 5 µg/m^3^ increase. | | | | | |
| HR: Hazard Ratios, CI: Confidence intervals, NO_2_: nitrogen dioxide, PM: particulate matter | | | | | |

**Table S5.** Associations between the exposures and invasive breast cancer, additionally adjusted for family history of breast and/or ovarian cancer.

| **Exposure** | **HR (95% CI)** |
| --- | --- |
| NO_2_ |  |
| First pregnancy | 1.02 (0.99-1.06)^a^ |
| Last pregnancy | 1.07 (1.02-1.11)^a^ |
| PM_10_ |  |
| First pregnancy | 1.06 (1.00-1.12)^a^ |
| Last pregnancy | 1.11 (1.04-1.18)^a^ |
| PM_2.5_ |  |
| First pregnancy | 1.04 (1.00-1.08)^b^ |
| Last pregnancy | 1.08 (1.03-1.12)^b^ |
| PM_coarse_ |  |
| First pregnancy | 1.07 (0.98-1.16)^b^ |
| Last pregnancy | 1.09 (1.00-1.19)^b^ |
| ^a^Per 10 µg/m^3^ increase. | |
| ^b^Per 5 µg/m^3^ increase. | |
| HR: Hazard Ratios, CI: Confidence intervals,  NO_2_: nitrogen dioxide, PM: particulate matter | |

**Table S6.** Associations between the exposures and invasive breast cancer, stratified by family history of breast and/or ovarian cancer.

|  | **Family history**^a, c^ | **No family history**^b, c^ |
| --- | --- | --- |
| **Exposure** | **HR (95% CI)** | **HR (95% CI)** |
| NO_2_ |  |  |
| First pregnancy | 1.01 (0.93-1.09)^d^ | 1.03 (0.99-1.07)^d^ |
| Last pregnancy | 1.07 (0.98-1.17)^d^ | 1.07 (1.02-1.12)^d^ |
| PM_10_ |  |  |
| First pregnancy | 1.05 (0.94-1.18)^d^ | 1.06 (1.00-1.13)^d^ |
| Last pregnancy | 1.13 (0.99-1.28)^d^ | 1.10 (1.03-1.18)^d^ |
| PM_2.5_ |  |  |
| First pregnancy | 1.03 (0.95-1.12)^e^ | 1.05 (1.00-1.09)^e^ |
| Last pregnancy | 1.10 (1.01-1.20)^e^ | 1.07 (1.02-1.12)^e^ |
| PM_coarse_ |  |  |
| First pregnancy | 1.08 (0.90-1.29)^e^ | 1.06 (0.97-1.17)^e^ |
| Last pregnancy | 1.09 (0.90-1.31)^e^ | 1.10 (0.99-1.21)^e^ |
| ^a^N_first pregnancy_ = 76 971; N_last pregnancy_ = 69 660. | | |
| ^b^N_first pregnancy_ = 664 232; N_last pregnancy_ = 562 931. | | |
| ^c^Models not adjusted for family history. | | |
| ^d^Per 10 µg/m^3^ increase. | | |
| ^e^Per 5 µg/m^3^ increase. | | |
| HR: Hazard Ratios, CI: Confidence intervals,  NO_2_: nitrogen dioxide, PM: particulate matter | | |

**Table S7.** Adjusted models for invasive breast cancer with interaction terms between the air pollutants at first pregnancy and 1) smoking, 2) family history of breast and/or ovarian cancer, and 3) education level.

|  | **NO_2_**^a^ | | **PM_10_**^a^ | | **PM_2.5_**^b^ | | **PM_coarse_**^b^ | |
| --- | --- | --- | --- | --- | --- | --- | --- | --- |
| **Stratum-specific effects of the air pollutants** | **HR** | **95% CI** | **HR** | **95% CI** | **HR** | **95% CI** | **HR** | **95% CI** |
| **Smoking** |  |  |  |  |  |  |  |  |
| Non-smokers | 1.04 | (1.01-1.08) | 1.09 | (1.04-1.15) | 1.06 | (1.03-1.10) | 1.12 | (1.04-1.21) |
| Smokers | 1.06 | (0.95-1.19) | 1.08 | (0.92-1.26) | 1.08 | (0.97-1.20) | 0.98 | (0.77-1.24) |
| **Family history** |  |  |  |  |  |  |  |  |
| No family history | 1.03 | (0.99-1.08) | 1.07 | (1.00-1.13) | 1.05 | (1.00-1.09) | 1.07 | (0.97-1.17) |
| Family history | 0.99 | (0.92-1.07) | 1.04 | (0.93-1.17) | 1.02 | (0.95-1.11) | 1.06 | (0.89-1.26) |
| **Education level** |  |  |  |  |  |  |  |  |
| Primary school up to 9 years | 1.08 | (0.93-1.26) | 1.23 | (1.00-1.51) | 1.12 | (0.98-1.29) | 1.43 | (1.06-1.94) |
| Secondary school | 1.06 | (1.01-1.12) | 1.11 | (1.02-1.20) | 1.08 | (1.02-1.14) | 1.09 | (0.97-1.22) |
| Postsecondary school | 1.03 | (0.99-1.07) | 1.07 | (1.01-1.14) | 1.05 | (1.01-1.10) | 1.10 | (1.00-1.22) |
| ^a^Per 10 µg/m^3^ increase. | | | | | | | | |
| ^b^Per 5 µg/m^3^ increase. | | | | | | | | |
| HR: Hazard Ratios, CI: Confidence intervals, NO_2_: nitrogen dioxide, PM: particulate matter | | | | | | | | |

**Table S8.** Associations between mean exposures during the last pregnancy period and breast cancer, by detection method, including all ages at diagnosis.

|  | **All invasive cases** n = (7397 cases/36 985 controls) | **Invasive cases  40-49 years of age**^a^ n = (5661 cases/28 305 controls) | **Screen-detected 40-49 years of age**^a^ n = (2382 cases/11 910 controls) | **All non-screen-detected** n = (4961 cases/24 805 controls) | **Non-screen-detected 40-49 years of age**^a^ n = (3256 cases/16 280 controls) |
| --- | --- | --- | --- | --- | --- |
| **NO_2_** |  |  |  |  |  |
| HR (95% CI)^b^ | 1.10 (1.04-1.15) | 1.09 (1.03-1.15) | 1.02 (0.93-1.12) | 1.13 (1.06-1.19) | 1.12 (1.05-1.21) |
| **PM_10_** |  |  |  |  |  |
| HR (95% CI)^b^ | 1.12 (1.05-1.21) | 1.11 (1.03-1.21) | 1.08 (0.95-1.23) | 1.14 (1.05-1.24) | 1.13 (1.02-1.26) |
| **PM_2.5_** |  |  |  |  |  |
| HR (95% CI)^c^ | 1.09 (1.04-1.14) | 1.08 (1.02-1.14) | 1.07 (0.99-1.17) | 1.09 (1.03-1.16) | 1.08 (1.01-1.16) |
| **PM_coarse_** |  |  |  |  |  |
| HR (95% CI)^c^ | 1.13 (1.02-1.24) | 1.12 (1.00-1.26) | 1.01 (0.84-1.21) | 1.18 (1.05-1.33) | 1.20 (1.03-1.40) |
| ^a^Includes cases diagnosed the year they turned 40. | | | | | |
| ^b^Per 10 µg/m^3^ increase. | | | | | |
| ^c^Per 5 µg/m^3^ increase. | | | | | |
| HR: Hazard Ratios, CI: Confidence intervals, NO_2_: nitrogen dioxide, PM: particulate matter | | | | | |

**Table S9.** Associations between the exposures and breast cancer, without pregnancy-associated breast cancer.

| **Exposure** | **HR (95% CI)** |
| --- | --- |
| NO_2_ |  |
| First pregnancy^c^ | 1.03 (1.00-1.06)^a^ |
| Last pregnancy^d^ | 1.05 (1.02-1.09)^a^ |
| 35 years of age^e^ | 1.03 (0.99-1.07)^a^ |
| PM_10_ |  |
| First pregnancy^c^ | 1.07 (1.02-1.12)^a^ |
| Last pregnancy^d^ | 1.10 (1.04-1.15)^a^ |
| 35 years of age^e^ | 1.07 (1.02-1.13)^a^ |
| PM_2.5_ |  |
| First pregnancy^c^ | 1.05 (1.02-1.08)^b^ |
| Last pregnancy^d^ | 1.06 (1.03-1.10)^b^ |
| 35 years of age^e^ | 1.05 (1.01-1.08)^b^ |
| PM_coarse_ |  |
| First pregnancy^c^ | 1.10 (1.02-1.18)^b^ |
| Last pregnancy^d^ | 1.11 (1.04-1.20)^b^ |
| 35 years of age^e^ | 1.10 (1.02-1.18)^b^ |
| ^a^Per 10 µg/m^3^ increase. | |
| ^b^Per 5 µg/m^3^ increase. | |
| ^c^n = 11 596 breast cancer cases. | |
| ^d^n = 11 436 breast cancer cases. | |
| ^e^n = 10 283 breast cancer cases. | |
| HR: Hazard Ratios, CI: Confidence intervals,  NO_2_: nitrogen dioxide, PM: particulate matter | |

**Table S10.** Associations between the exposures and breast cancer, without cases diagnosed within one year after start of follow-up.

| **Exposure** | **HR (95% CI)** |
| --- | --- |
| NO_2_ |  |
| First pregnancy^c^ | 1.03 (1.00-1.06)^a^ |
| Last pregnancy^d^ | 1.05 (1.02-1.09)^a^ |
| 2 years after last delivery^e^ | 1.07 (1.02-1.11)^a^ |
| 35 years of age^f^ | 1.03 (0.99-1.06)^a^ |
| PM_10_ |  |
| First pregnancy^c^ | 1.08 (1.03-1.13)^a^ |
| Last pregnancy^d^ | 1.10 (1.04-1.15)^a^ |
| 2 years after last delivery^e^ | 1.10 (1.04-1.17)^a^ |
| 35 years of age^f^ | 1.07 (1.01-1.13)^a^ |
| PM_2.5_ |  |
| First pregnancy^c^ | 1.05 (1.02-1.08)^b^ |
| Last pregnancy^d^ | 1.06 (1.03-1.10)^b^ |
| 2 years after last delivery^e^ | 1.07 (1.03-1.12)^b^ |
| 35 years of age^f^ | 1.04 (1.01-1.08)^b^ |
| PM_coarse_ |  |
| First pregnancy^c^ | 1.10 (1.03-1.18)^b^ |
| Last pregnancy^d^ | 1.11 (1.04-1.20)^b^ |
| 2 years after last delivery^e^ | 1.10 (1.02-1.19)^b^ |
| 35 years of age^f^ | 1.09 (1.01-1.17)^b^ |
| ^a^Per 10 µg/m^3^ increase. | |
| ^b^Per 5 µg/m^3^ increase. | |
| ^c^n = 11 950 breast cancer cases. | |
| ^d^n = 11 436 breast cancer cases. | |
| ^e^n = 9970 breast cancer cases. | |
| ^f^n = 10 207 breast cancer cases. | |
| HR: Hazard Ratios, CI: Confidence intervals,  NO_2_: nitrogen dioxide, PM: particulate matter | |

**Table S11.** Associations between NO_2_ and PM_10_ exposure and invasive breast cancer, stratified by time periods for first and last delivery (1991-1995, 1996-2000, and 2001-2005).

|  | **Group 1 (1991-1995)**^a^ | **Group 2 (1996-2000)**^b^ | **Group 3 (2001-2005)**^c^ |
| --- | --- | --- | --- |
| **NO_2_** |  |  |  |
| **First pregnancy** |  |  |  |
| HR (95% CI)^d^ | 0.98 (0.88-1.10) | 1.06 (0.95-1.19) | 1.10 (1.00-1.21) |
| **Last pregnancy** |  |  |  |
| HR (95% CI)^d^ | 1.01 (0.90-1.14) | 1.10 (0.98-1.24) | 1.12 (1.01-1.24) |
| **PM_10_** |  |  |  |
| **First pregnancy** |  |  |  |
| HR (95% CI)^d^ | 1.04 (0.89-1.23) | 1.11 (0.94-1.32) | 1.11 (0.96-1.28) |
| **Last pregnancy** |  |  |  |
| HR (95% CI)^d^ | 1.09 (0.93-1.29) | 1.17 (0.98-1.40) | 1.15 (0.99-1.34) |
| ^a^First pregnancy: N = 90 870, n cases = 921; Last pregnancy: N = 90 816, n cases = 921. | | | |
| ^b^First pregnancy: N = 67 024, n cases = 778; Last pregnancy: N = 67 000, n cases = 777. | | | |
| ^c^First pregnancy: N = 77 264, n cases = 1040; Last pregnancy: N = 77 246, n cases = 1040. | | | |
| ^d^Per 10 µg/m^3^ increase. | | | |
| HR: Hazard Ratios, CI: Confidence intervals, NO_2_: nitrogen dioxide, PM: particulate matter | | | |
